# Ultrasound-activated ciliary bands for microrobotic systems inspired by starfish

**DOI:** 10.1038/s41467-021-26607-y

**Published:** 2021-11-09

**Authors:** Cornel Dillinger, Nitesh Nama, Daniel Ahmed

**Affiliations:** 1 Acoustic Robotics Systems Lab, Institute of Robotics and Intelligent Systems, Department of Mechanical and Process Engineering, ETH Zurich, Zurich, Switzerland; 2 Department of Mechanical and Materials Engineering, University of Nebraska-Lincoln, Lincoln, NE USA

**Keywords:** Mechanical engineering, Acoustics

## Abstract

Cilia are short, hair-like appendages ubiquitous in various biological systems, which have evolved to manipulate and gather food in liquids at regimes where viscosity dominates inertia. Inspired by these natural systems, synthetic cilia have been developed and utilized in microfluidics and microrobotics to achieve functionalities such as propulsion, liquid pumping and mixing, and particle manipulation. Here, we demonstrate ultrasound-activated synthetic ciliary bands that mimic the natural arrangements of ciliary bands on the surface of starfish larva. Our system leverages nonlinear acoustics at microscales to drive bulk fluid motion via acoustically actuated small-amplitude oscillations of synthetic cilia. By arranging the planar ciliary bands angled towards (+) or away (−) from each other, we achieve bulk fluid motion akin to a flow source or sink. We further combine these flow characteristics with a physical principle to circumvent the scallop theorem and realize acoustic-based propulsion at microscales. Finally, inspired by the feeding mechanism of a starfish larva, we demonstrate an analogous microparticle trap by arranging + and − ciliary bands adjacent to each other.

## Abstract (web summary)

Most engineered ciliary bands generate simple hydrodynamics with unidirectional flow profiles. Here, inspired by the starfish larva, the authors show acoustically-activated microrobots with cilia that can control direction of liquid flow through oscillations, exhibiting propulsion at microscales.

## Introduction

Cilia are short, hair-like appendages present in numerous biological living systems. They can be found on the surfaces of many organisms, including algae and invertebrate larvae, which are naturally evolved to manipulate and gather food in liquids, where viscosity dominates inertia. Ciliated surfaces are also present in most mammals’ respiratory tracts, where they trap and move particulates towards the nostrils, and in Fallopian tubes, where they transport the ovum towards the uterus. Inspired by nature’s cilia and their functions, engineered synthetic cilia and ciliary bands are of great interest for lab-on-chip devices and microrobotic systems. In particular, synthetic cilia promise solutions for many fundamental functions including propulsion, liquid pumping and mixing, and particle manipulation—all difficult to realize at microscale due to the lack of inertia.

Artificial cilia are commonly driven by external fields, such as electric, magnetic, light, and pressure fields. Magnetically-driven cilia, in particular, have become popular due to their relatively simple and easy operation. For example, the motion of artificial cilia has been realized with the application of a handheld magnet to composite magnetic-polymeric nanorod arrays^1^. In another method, a chain of magnetism-based self-assembled cilia is first formed through the dipole–dipole interaction of superparamagnetic particles immersed in liquid and subsequently induced to rotate via a rotating magnetic field^2^. Alternative approaches implemented for fluid pumping and particle transport^3^ include electrostatically-activated cilia created with metal-coated polymeric films for mixing applications at low Reynolds number; light-driven cilia fabricated using azo-benzene-doped liquid crystals^4^; and cilia containing multiple pneumatically-controlled actuators^5^. Consequently, there is a growing interest in understanding the collective behavior of multiple neighboring cilia, i.e., ciliary arrays. Recently, magnetic ciliary carpets have shown to exhibit metachronal waves, which feature has been used to transport fluid^6,7^. However, most studies of the collective behavior of multiple neighboring cilia have been numerical in focus^8–10^, and most engineered ciliary bands generate simple hydrodynamics with unidirectional flow profiles regardless of whether individual cilia undergo phase shift on a flat or curved ciliary array. As of yet, diversity in the forms of ciliary bands and their corresponding intricate hydrodynamic functions remain to be explored. Nature’s swimmers provide ready inspiration; for example, marine invertebrate larvae, such as those of *Patiria miniate* (starfish), can adjust the orientation of cilia in their ciliary bands (i.e., densely packed cilia) to control the direction of liquid flow, developing an analogous fluid source and sink and exploiting them for propulsion and feeding^11^. Taking the natural arrangements of the cilia on the surface of starfish larva as our inspiration, we developed ultrasound-activated ciliary bands.

While several different approaches have been developed to exploit artificial cilia for biomedical applications, acoustically-activated cilia have received little attention^12^—a surprising trend given the widespread use of ultrasound in microfluidics and biomedical applications. In particular, while the acoustic vibration of individual synthetic cilia has been employed for liquid pumping^13^, the interaction of multiple cilia in close proximity, i.e., in ciliary bands (as observed in biological systems), remains largely unexplored. Cilia and ciliary bands activated by ultrasound are particularly attractive because ultrasound is safe to most biological systems, noninvasive, and penetrates deep in the body of an animal model. Ultrasound-based ciliary bands promise to be an extremely versatile tool for various lab-on-chip applications and developing micro- and nanorobots.

Here, we demonstrate ultrasound-activated synthetic ciliary bands, which undergo small-amplitude oscillation and produce controlled liquid flow upon their activation. When the ultrasound-actuated planar ciliary bands are angled toward each other (+) on a supporting surface, the fluid is pushed away from the surface, mimicking a flow source. In contrast, when the cilia are angled away from each other (−), the liquid is forced in toward the surface, mimicking a flow sink. We leverage nonlinear acoustics in conjunction with these source/sink arrangements to develop a design principle of propulsion for acoustic-based microrobots. Using the concept of source and sink, we demonstrate directionality. Finally, by arranging + and − ciliary bands adjacent to each other, we engineer a novel microparticle transport and trapping mechanism that mimics the feeding mechanism of starfish larva.

## Results

### Bioinspired ciliary bands

Inspired by the remarkable natural arrangements of ciliary bands on the surface of invertebrate larvae, we developed new designs for ultrasound-based ciliary bands that leverage the same physical principles. To validate our concept, we fabricated + and − arrangements of cilia using the ultraviolet (UV) photopolymerization method and performed experiments to characterize their behavior in an acoustic field. The UV photopolymerization method was developed under an inverted microscope. In short, masks containing the ciliary band designs were placed at the field stop of the microscope. UV light passes through a 20x objective and polymerized the polyethylene glycol and photo-initiator solution sandwiched between two glass slides (Supplementary Fig. 1). The fabricated + and − ciliary bands comprised two to eight cilia on each side. Each cilium had a length, base thickness, and height of *L* ≈ 100 μm, *W* ≈ 15–35 μm, and *H* ≈ 50 μm (Supplementary Fig. 2), respectively, and as a set were arranged in series with separation of 20–40 μm. This method enables a one-step high-throughput fabrication of microarchitectures. After fabrication, the ciliary bands were placed in an acoustic chamber filled with a liquid solution containing tracer particles. A piezo transducer, which generated the acoustic field, was bonded next to the acoustic chamber and connected to an electronic function generator (Supplementary Fig. 3). A combination of transverse acoustic waves from the glass slide and a longitudinal wave propagating into the liquid activates the microstructure. The entire setup was mounted on an inverted microscope, and experimental results were captured using light-sensitive and high-speed cameras.

**Fig. 1:**
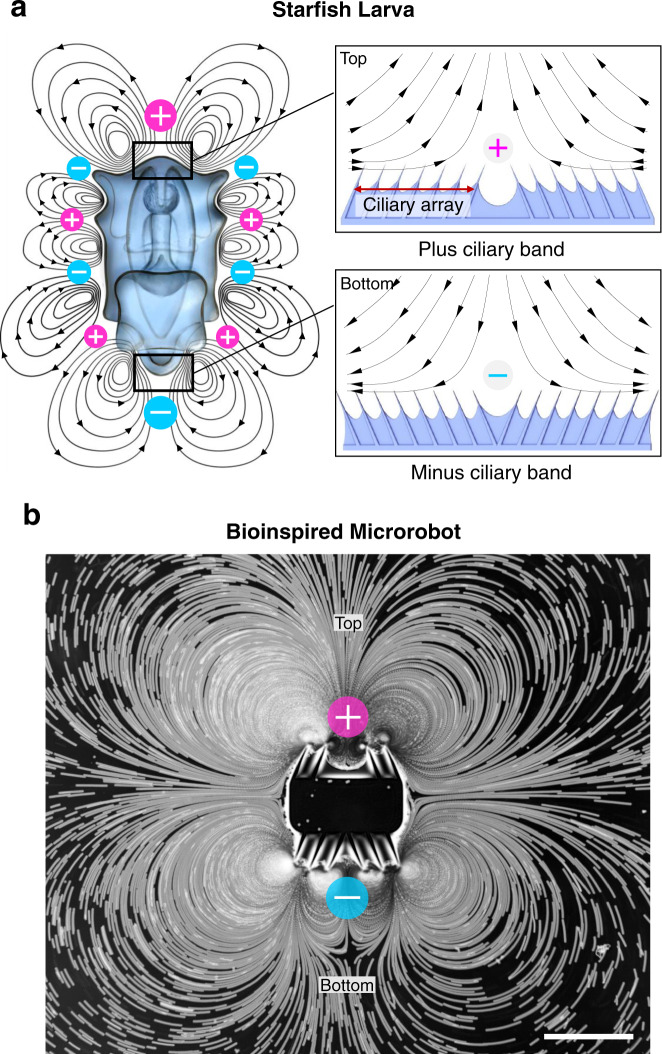
Starfish larva-inspired ultrasound ciliary band designs. **a** A starfish larva exhibits a complex flow profile of counter-rotating vortices generated by a series of + and − ciliary bands arranged on its body protuberances. Inset: (top) A + ciliary band comprises a pair of angled ciliary arrays that face each other and causes the liquid to flow away on a plane perpendicular to the center of the ciliary band. (Bottom) A − ciliary band consists of two ciliary arrays oriented away from each other and directs the liquid flow to the center of the band. **b** A starfish-inspired microrobot consisting of a + (top) and a − (bottom) ciliary band placed in a tracer solution. The ciliary bands oscillate when actuated by ultrasound, producing complex flow profiles similar to those observed with its biological counterpart. Scale bar, 250 μm.

Over the course of the experiment, the acoustic field’s excitation frequency was modulated from 20–100 kHz while maintaining an applied power of 1–25 V peak-to-peak (V_PP_). When exposed to such an acoustic field, the ciliary band oscillate in-phase and undergoes small-amplitude oscillations (see Supplementary Fig. 4 and Supplementary Movie 1). These oscillations result in a time-averaged steady flow field in the surrounding liquid, also referred to as acoustic streaming^14^. This acoustic streaming is driven by the dissipation of acoustic energy flux inside the fluid system that occurs, in general, both within and outside of the viscous boundary layer. However, for devices where the characteristic dimensions are much smaller compared to the acoustic wavelength (L ≪ λ), the acoustic streaming is primarily driven by the viscous dissipations within the boundary layer^15,16^. For the cilia system considered in this work, the thickness of the viscous boundary layer can be computed as $\delta =\sqrt{\nu /(\pi f)}=\; \sim\! 1.8-4.0\upmu {{{{{\rm{m}}}}}}$, where $\nu \approx {10}^{-6}\,{{{{{{\rm{m}}}}}}}^{2}/{{{{{\rm{s}}}}}}$ is the kinematic viscosity of water at room temperature, and *f* = 20–100 kHz is the excitation frequency. The viscous dissipations in this thin boundary layer drive the outer bulk streaming motion. This streaming profile is determined by the arrangement of the ciliary array relative to the normal axis of the underlying bulk surface. Referring to Fig. 1a insets, a + ciliary band comprises a pair of ciliary arrays that face each other and causes the liquid to flow away on a plane perpendicular to the center of the ciliary band, analogous to a fluid source. Conversely, a − ciliary band consists of two ciliary arrays oriented away from each other and directs the streamlines of the liquid flow to the center of the band, analogous to a fluid sink. Fig. 1b represents the complex flow profile of a bioinspired microrobot by combining + and − ciliary bands.

**Fig. 2:**
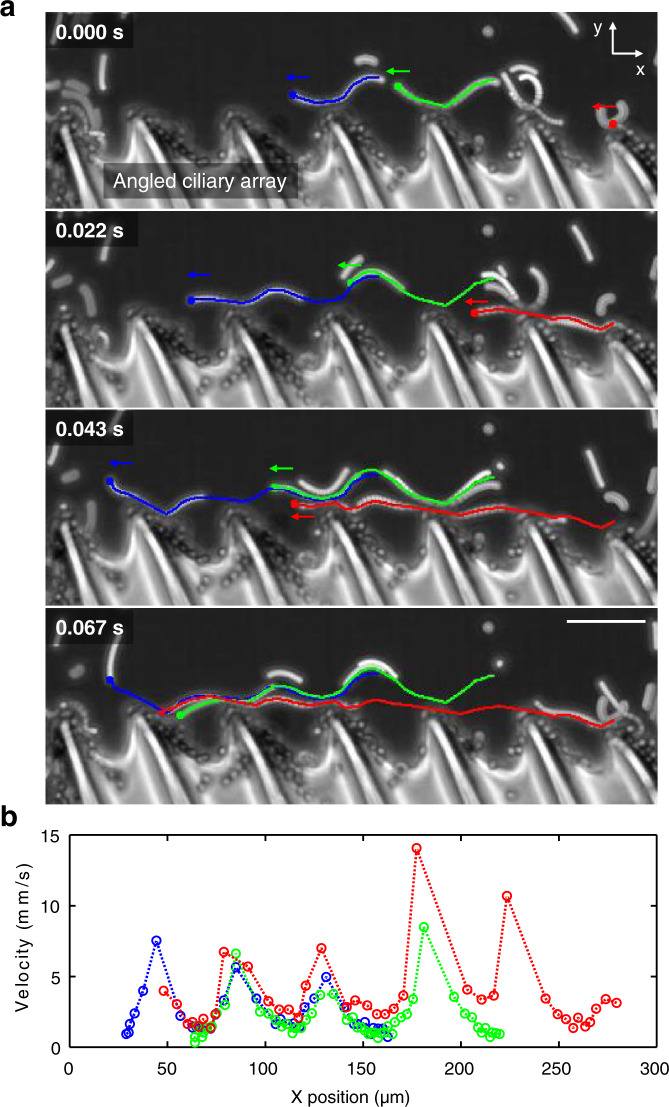
Tangential flow along angled ciliary array when exposed to ultrasound. **a** Image sequences (at *t* = 0.000–0.067 s) demonstrate ~6 μm tracer particles, indicated by red, green, and blue lines, traveling along one cilium tip to the next from right-to-left in the direction the tips are angled at excitation frequency and amplitude of 33.7 kHz and 5 V_PP_, respectively (see also Supplementary Movie 2). Due to residual non-polymerized and sticky polymer material on the surface of individual cilia and in between located web-like structures, tracers adhere to the structure’s surface. **b** Velocity analysis of the tracers (Source Data 1 and Supplementary Software 1), along the horizontal axis of the image sequences in **a**, revealed a cyclic acceleration/deceleration pattern. Particles reached maximum speeds when they approached a ciliary tip, followed by a deceleration phase. Scale bar, 50 μm.

**Fig. 3:**
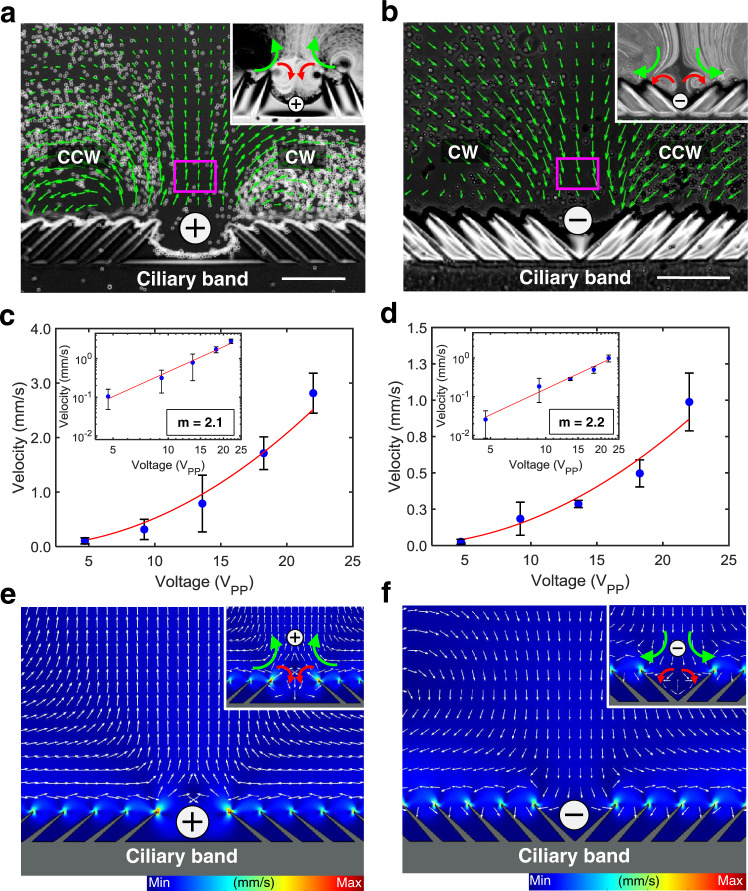
Experimental, characterization, and numerical demonstration of bioinspired ultrasound ciliary bands (Supplementary Movie 4). **a** PIV generated velocity fields of a + ciliary band that causes the liquid to flow away on a plane perpendicular to the center of the ciliary band at excitation frequency and amplitude of 68.7 kHz and 20 V_PP_, respectively. The inset indicates localized counter-rotating vortices at the innermost tips of the + ciliary band configuration. **b** PIV generated velocity fields of a − ciliary band, which directs the liquid flow to the center of the band at 68.8 kHz and 22 V_PP_, respectively. The inset indicates localized counter-rotating vortices at the innermost tips of the − ciliary band configuration. Plots of average velocities of **c**. + and **d**. − ciliary bands at sites indicated by magenta boxes in **a** and **b** versus voltage applied. The particles immersed in water transport at a speed proportional to the square of the voltage applied and this quadratic relation is reasonably well satisfied as indicated by the log plots. In each data point, 150−400 velocity measurements are averaged and the standard deviation is calculated and they are represented as black error bars (Source Data 2 and Supplementary Software 2). Numerical simulation of microstreaming using perturbation approach of **e** + ciliary band and **f** − ciliary band. The insets indicate numerically calculated counter-rotating vortices at the innermost tips of the respective ciliary band configuration. Color bars represent normalized streaming velocities. Scale bar, 100 μm.

To understand the behavior of a + ciliary band, we first looked at particle transport in the right half array of the + ciliary band when exposed to ultrasound. The tracer particles, as indicated by red, green, and blue trajectories in Fig. 2a, rapidly hop from the tip of one cilium to another, with particles achieving velocities as high as 10 mm/s when they approach the tip. The ciliary array’s direction dictates the flow direction, i.e., the flow is guided from right to left along with the right ciliary array (Fig. 2b and Supplementary Movie 2). The rapid motion of particles along that array produces a clockwise (CW) vortex in the right half of the ciliary band, which can be understood through the large velocity differential between the fast-moving tracers along the surface of the ciliary array and the low-velocity tracers having passed the last cilium (Supplementary Fig. 5 and Supplementary Movie 3). Similarly, fluid flow along the left half produces a counter-clockwise (CCW) vortex. Thus, an analogous fluidic source is formed due to the build-up of the two counter-rotating vortices, producing an outward flow at the center of the + ciliary band, as shown in Fig. 3a. Figure 3b likewise illustrates the liquid flow field near a − ciliary band. In contrast to Fig. 3a, the flow field for a − ciliary band is analogous to a fluid sink and produces an inward flow at the center (see also Supplementary Movie 4). In a control experiment, when a straight ciliary array was exposed to an acoustic field, we observed no significant particle transport or fluid flow from one cilium to another (Supplementary Fig. 6 and Supplementary Movie 5). Instead, the tracer particles were trapped in small vortices around each cilia tip. Therefore, an angulated ciliary array is responsible for the tangential streaming in the direction of the cilia tips, which is critical to developing the acoustic analogous source and sink.

The strength of the flow produced from + and − ultrasound ciliary bands is determined by the intensity of the ambient acoustic field, which is controlled by adjusting the voltage applied to the piezoelectric transducer. Specifically, the streaming is driven by force and mass sources that depend quadratically on the first-order pressure and velocity, which in turn, depend linearly on the applied displacement amplitude^16^. Furthermore, prior numerical and experimental investigations have revealed that, for small values of signal power, the displacement amplitude depends linearly on the applied voltage^16,17^. Consequently, the streaming is expected to scale quadratically with the applied voltage (i.e., streaming velocity $\propto \,{V}_{PP}^{\,2}$). To investigate this scaling, we employed particle image velocimetry (PIV)^18^ to measure the average velocities normal to the ciliary bands at sites indicated by magenta boxes in Fig. 3a, b. Figures 3c, d show that this quadratic relation is reasonably well satisfied by the ciliary bands. We note that the vertical streaming velocity of the + ciliary band is larger compared to the − configuration; likely due to the closer placement of the innermost cilia tips of the + ciliary band, which leads to each half band contributing more to the vertical streaming.

We reproduced the streaming flow patterns via numerical simulations based on the established perturbation approach^16,19^. Briefly, the perturbation approach expresses the fluid response to acoustic actuation as a sum of the first-order harmonic fields (v_1_, *p*_1_, *ρ*_1_) and the second-order steady fields (v_2_, *p*_2_, *ρ*_2_), e.g., ${{{{{\bf{v}}}}}}=\varepsilon {{{{{{\bf{v}}}}}}}_{1}+{\varepsilon }^{2}{{{{{{\bf{v}}}}}}}_{2}+\ldots ,$ where *ε* is an appropriate smallness parameter^19^. This approach represents the steady flow field induced by the ciliary bands of the microrobot in a fluid by a second-order system of equations (v_2_, *p*_2_, *ρ*_2_), which, in turn, is driven by the body force and mass source terms stemming from nonlinear interactions of the time-harmonic first-order fields (v_1_, *p*_1_, *ρ*_1_). Further details of the theoretical and numerical formulation can be found in Supplementary Notes. The numerical simulations yielded the same qualitative flow patterns (Fig. 3e, f) as the experimental results and therefore can serve as a useful tool in assessing the flow patterns for different configurations of ciliary arrays.

Engineering ciliary bands did not show any evidence of resonance characteristics. Nonetheless, we observed the frequency-dependent behavior of our system. Specifically, we observed that the performance of the system, with regards to the generation of streaming, was highest at 68.5 kHz. This can be attributed to the fact that the piezo transducer’s oscillation amplitude coupled with the glass slide is maximum at this frequency, which is likely to be one of the resonances of the piezo/glass system, see also Supplementary Fig. 7. The vibration of the acoustic ciliary bands is the primary driver of the resulting flow field. While direct measurement of this vibration is difficult to realize in experiments, our results indicate that the flow field around the swimmer is rather independent of the orientation of the swimmer with respect to the direction of the background acoustic field (Supplementary Fig. 8). Our theoretical analysis reveals that the relative strength of the forcing terms with respect to the viscous terms in the second-order equations is determined by the frequency Reynolds number, ${{{{{\mathrm{Re}}}}}}_{f}=2\pi f\varDelta sL/\nu$, where Δ*s* is the oscillation amplitude, and the separation of time and length scales between the acoustic actuation and streaming flow (see also Eq. (4)). Therefore, these ciliary bands can generate motion as long as their oscillation frequency is sufficiently high to introduce inertia in the second-order equations.

### Bioinspired microrobot

Microrobots could facilitate specialized tasks in medicine^20^, including surgical procedures and drug delivery to hard-to-access sites in vivo. Chemical^21–29^ and external field-driven, such as magnetic^30–38^, light^39–41^, electrical^42^, biohybrid^43–45^, or ultrasound^46–52^—microrobots are attractive because they do not require an onboard power supply or intricate moving parts and allow wireless control of the microrobot. Nonlinear ultrasound provides an alternate and attractive method to generate propulsion in vivo. Most extant ultrasound-based swimmers utilize single cilia^48,53^; in contrast, our starfish-inspired swimmer incorporates ciliary arrays and bands, thus making our method of propulsion more robust. This robot design is more stable than bubble-based microswimmers, as a microbubble can grow over time and therefore not entirely stable^47,54^. In this section, we analyse the swimming motion of our bioinspired microrobot. The applied ultrasound has a wavelength on the order of a centimeter (~2.2 cm), which is an order of magnitude larger than the microrobot (~280 μm). As a result, the swimmer is subjected to uniform pressure on all sides. This expectation is further corroborated by a control experiment performed on a swimmer lacking ciliary bands and under acoustic actuation of different frequencies and amplitudes (Supplementary Fig. 9); the lack of resultant motion of the device suggests (i) a nearly-uniform acoustic pressure field, with no significant net force on the swimmer and (ii) the radiation force of the acoustic wave from the piezo transducer does not contribute to any motion.

**Fig. 4:**
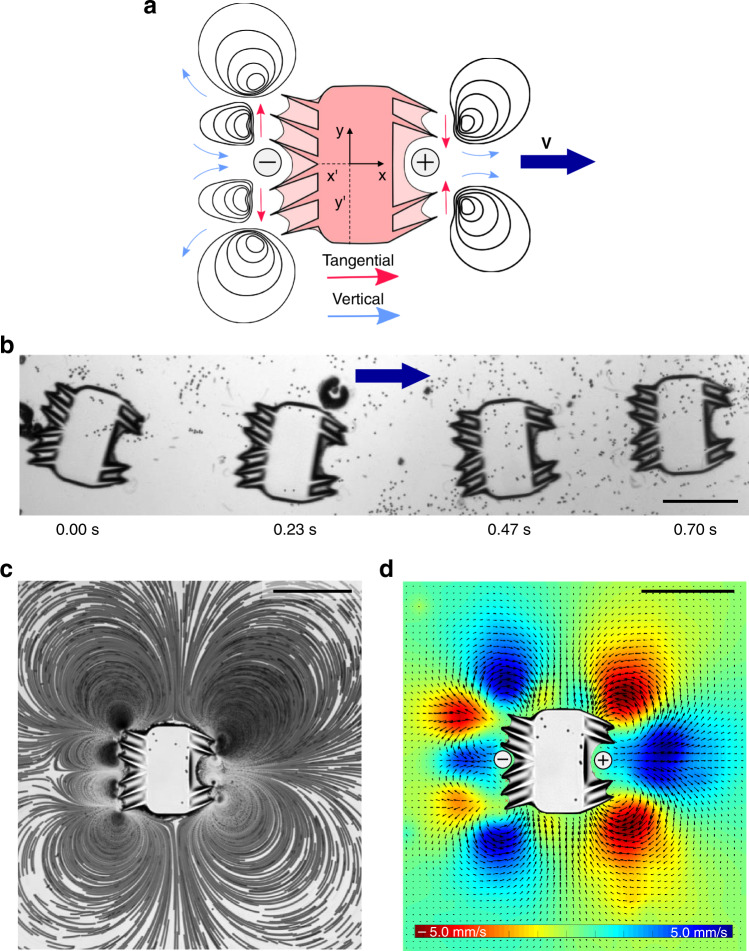
Propulsion of bioinspired ultrasound microrobot. **a** Schematic of an artificial microrobot consisting of a – ciliary band on the left and a + ciliary band on the right. Red and blue arrows indicate tangential and vertical velocity, respectively. **b** Superimposed time-lapse image (time is advancing in the direction of the blue arrow at the values given below) of controlled translation motion of a microrobot at excitation frequency and amplitude of 68.8 kHz and 20 V_PP_, respectively (see also Supplementary Movie 6). **c** Image sequence illustrating the streaming flow profile of the bioinspired microrobot. A stack of 150 images was used; the video was captured using a high-speed camera at a framerate of 1069 fps (see also Supplementary Movie 7). **d** PIV generated velocity fields demonstrate the complex flow behavior developed in the surrounding liquid. Minimum and maximum flow velocities are marked by red and blue regions, respectively. Scale bars, 250 μm.

We designed + and − ciliary bands on the left and right sides, respectively, of the soft robot, as shown in the schematic in Fig. 4a. They were released from the glass slide by lifting the top coverslip from one side using a pair of needles and carefully pushing the microrobots bulk body with a needle tip avoiding surfaces that contained ciliary arrays. As the microrobot was exposed to ultrasound of 68.8 kHz at 20 V_PP_, it exhibited left-to-right translational propulsion (along the direction of the short axis). Figure 4b demonstrates an image sequence depicting the microrobot traveling at ~2.6 mm/s (10 body lengths per second; Supplementary Movie 6). Similar to ultrasound ciliary bands, the microrobotic design did not exhibit any resonance characteristics. As long as the amplitude of the acoustic field was sufficiently high at a given voltage, the microrobot should propel. The trajectory of the microrobot, i.e., translating left-to-right (along the direction of the short axis), is further dictated by the configuration of ciliary bands. Figure 4c, d shows the complex fluid flow profile produced by the microrobot, comprised of a series of CW and CCW vortices. As expected, the arrangement of those vortices is symmetrical across the short axis (*xx*′), of the swimmer. Consequently, the forces exerted by the fluid on the swimmer along the long axis (*yy*′) was balanced, resulting in a net forcing and concomitant propulsion solely along the short axis (see also Supplementary Movie 7).

The Reynolds number of the bioinspired microrobot can be estimated as $Re=uD/\nu \approx 0.7$, where *μ* = 2.6 mm/s is the swimming velocity and *D* = 280 μm is the width of the microrobot. This value also suggests that the system is viscous-dominated and thus must not exhibit any noticeable motion, per the scallop theorem. However, since ultrasound causes the ciliary band to oscillate, we must consider the Reynolds number associated with the oscillation of the cilia. For ultrasound-actuated systems, the steady flow field is driven by the nonlinear interactions of the harmonic first-order response of the fluid. This can also be deduced from the perturbation expansion approach where the second-order system of equations is driven by time-averaged nonlinear first-order terms that scale with *Re*_*f*_ (see also Supplementary Notes). Depending on *Re*_*f*_, these first-order response-dependent forcing terms (the second and third term on the left-hand side of Eq. S7 in Supplementary Notes) can introduce sufficient inertia in the system to achieve swimming, even for reciprocal motion, rendering the Scallop theorem inapplicable.

The first-order fluid response is represented by the linearized Navier–Stokes equations, (Eq. S4 and S5) and can be expressed in terms of the nondimensional frequency Reynolds number. We begin by defining the following nondimensional quantities:

1 $$\hat{\rho }=\frac{{\rho }_{0}}{\tilde{\rho }},\,{\hat{{{{{{\bf{v}}}}}}}}_{1}=\frac{{{{{{{\bf{v}}}}}}}_{1}}{\tilde{v}},\,\hat{t}=\frac{t}{\tilde{t}},\,\hat{\mu }=\frac{\mu }{\tilde{\mu }},$$

where $\tilde{\rho },\tilde{v},\tilde{t}$, and $\tilde{\mu }$ denote the characteristic scales for the first-order velocity, density, time, and viscosity, respectively. Using these nondimensional quantities, the first-order momentum equation can be expressed as

2 $$R{e}_{f}\hat{\rho }\frac{\partial {\hat{{{{{{\bf{v}}}}}}}}_{1}}{\partial \hat{t}}=-\hat{\nabla }{\hat{p}}_{1}+\hat{\mu }{\hat{\nabla }}^{2}{\hat{{{{{{\bf{v}}}}}}}}_{1}+({\hat{\mu }}_{b}+\frac{1}{3}\hat{\mu })\hat{\nabla }(\hat{\nabla }.{\hat{{{{{{\bf{v}}}}}}}}_{1}),$$

where, as expected, the relative strength of the inertial and viscous terms at the first-order is characterized by the frequency Reynolds number given as $R{e}_{f}=\frac{\tilde{\rho }\tilde{v}\tilde{L}}{\tilde{\mu }}$, with $\tilde{L}$ being the characteristic length scale for the first-order system. Noting that the first-order (actuation) velocity scale can be expressed as $\tilde{v}=\omega \varDelta s=2\pi f\varDelta s$ and denoting kinematic viscosity as $\nu =\frac{\tilde{\mu }}{\tilde{\rho }}$, the frequency Reynolds number can be expressed as ${{{{{\mathrm{Re}}}}}}_{f}=2\pi f\varDelta sL/\nu .$

Next, we consider the relative strength of the forcing term in the second-order momentum equation (Eq. (3)) with respect to the viscous terms.

3 $${\rho }_{0}\left\langle \frac{\partial {{{{{{\bf{v}}}}}}}_{2}}{\partial t}\right\rangle +\left\langle {\rho }_{1}\frac{\partial {{{{{{\bf{v}}}}}}}_{1}}{\partial t}\right\rangle +{\rho }_{0}\langle ({{{{{{\bf{v}}}}}}}_{1}.\nabla ){{{{{{\bf{v}}}}}}}_{1}\rangle =-\nabla {p}_{2}+\mu {\nabla }^{2}{{{{{{\bf{v}}}}}}}_{2}+\left({\mu }_{b}+\frac{1}{3}\mu \right)\nabla (\nabla .{{{{{{\bf{v}}}}}}}_{2})$$

Specifically, the third term on the left-hand side of Eq. (3) scales as ${\rho }_{0}\langle ({{{{{{\bf{v}}}}}}}_{1}.\nabla ){{{{{{\bf{v}}}}}}}_{1}\rangle \sim \frac{\tilde{\rho }{\tilde{v}}^{2}}{\tilde{L}}$, while the viscous term in Eq. (3) scales *as* $\mu {\nabla }^{2}{{{{{{\bf{v}}}}}}}_{2} \sim \frac{\tilde{\mu }{\tilde{v}}_{s}}{{\tilde{L}}_{s}^{2}}$. Therefore, the relative strength of the forcing term with respect to the viscous terms is given by the ratio

4 $$\frac{\tilde{\rho}\tilde{v}^{2}}{\tilde{L}}\cdot \frac{\tilde{L}^{2}_{s}}{\tilde{\mu}\tilde{v}_{s}}\sim Re_{f}\frac{\tilde{v}}{\tilde{v}_{s}}\left(\frac{\tilde{L}_{s}}{\tilde{L}}\right)^{2} \sim Re_{f}\frac{\tilde{v}_{s}}{\tilde{v}}\left(\frac{\tilde{t}_{s}}{\tilde{t}}\right)^{2}$$

where ${\tilde{v}}_{s},{\tilde{L}}_{s},$ and ${\tilde{t}}_{s}$ denote the characteristic scales for the streaming velocity, length, and time, respectively. Thus, the forcing term in the second-order system depends on the frequency Reynolds number as well as the separation of time and length (or velocity) scales between the first-order and the streaming flow. Consequently, a sufficiently large Reynolds number and adequately separated time and length scales can introduce significant inertia in the second-order equations and therefore violate the contingency of the scallop theorem, to achieve propulsion even with a reciprocal actuation. Furthermore, since *Re*_*f*_ scales linearly with the frequency and amplitude of a vibrating object, the frequency Reynolds number of the ciliary bands can be engineered to have intermediate values, which implies that inertia can be introduced to produce propulsion at microscale even for reciprocal motion. The frequency Reynolds number of our oscillating cilium can be estimated as $Re_{f} = 2\pi f\Delta sL/v \approx 31.4-62.8$, where *f* = 100 kHz is the excitation frequency and $\varDelta s\approx 0.5-1.0\,\upmu m$ (see also Supplementary Fig. 4 and Supplementary Movie 1).

Our control experiments (Supplementary Fig. 9) reveal that a system without any cilia does not result in any net propulsion, indicating that the background acoustic field does not result in any significant propulsive force. In contrast, when a ciliary band is introduced, the interaction of the ciliary band with the acoustic wave results in two phenomena: (a) development of a perturbed (first-order) acoustic field and (b) development of a (second-order) streaming field arising from the nonlinear interactions of this perturbed acoustic field. Both these phenomena result in a net propulsive force on the swimmer at the second-order level. Therefore, the propulsion of the swimmer can be understood as a direct consequence of the presence of ciliary arrays that results in a net propulsive force given as

5 $${{{{{{\bf{F}}}}}}}_{{{{{{\bf{A}}}}}}}=\oint_{\partial {\varOmega }_{1}}\langle {{{{{{\boldsymbol{\sigma }}}}}}}_{2}\rangle \cdot {{{{{\bf{n}}}}}}dA+\oint_{\partial {\varOmega }_{1}}{\rho }_{0}\langle {{{{{{\bf{v}}}}}}}_{1}{{{{{{\bf{v}}}}}}}_{1}\rangle \cdot {{{{{\bf{n}}}}}}dA$$

where $\partial {\varOmega }_{1}$ represents the surface of the microrobot. Here, the first term accounts for the contribution from the stress 〈**σ**_2_〉 developed by localized acoustic microstreaming, while the second term represents the nonlinear interactions of the first-order harmonic velocity response.

A comprehensive characterization of this propulsive force represents significant challenges, both from an experimental as well as a numerical perspective. However, the overall flow profile around the microrobot can be viewed as a combination of flow profiles generated by several individual cilia. Eq. (S8) and (S9) in Supplementary Notes show the expressions for the first and second-order velocity fields around a single cilium, which can be observed to depend both on the acoustic actuation amplitude and the operational frequency. These expressions are instructive of the frequency and amplitude scaling governing the flow field around a microrobot. Consequently, microrobot propulsion can be achieved by tuning these operational parameters to invalidate the contingency of the scallop theorem by introducing sufficient inertia in the system.

### Microparticle transport

**Fig. 5:**
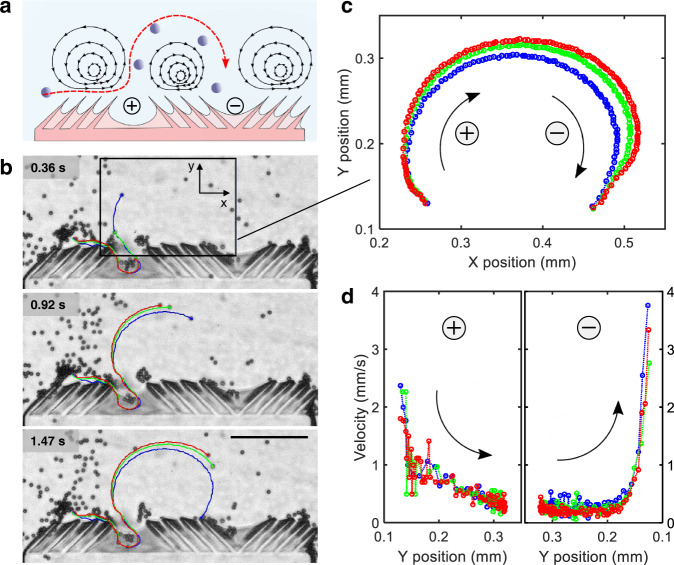
Bioinspired microparticle trapping using a combination of + and – ciliary bands. **a** Schematic of a bioinspired trapping mechanism consisting of a + ciliary band adjacent to a – ciliary band (see also Supplementary Movie 8). **b** Image sequence demonstrating microparticles becoming trapped in the – ciliary band, as indicated by red, green, and blue trajectories at 68.5 kHz and 20 V_PP_. **c** Expanded spatial trajectories of microparticles that became trapped by the flow field produced from the adjacent + and – ciliary bands. **d** Plots of microparticle velocity versus y-position: the left panel indicates the source and the right panel the sinking behavior of the trapping ciliary band (Source Data 3 and Supplementary Software 3). Scale bar, 200 μm.

Next, we introduce a microparticle trapping strategy inspired by the feeding mechanism of a starfish larvae^11^. Briefly, this mechanism is characterized by the juxtaposition of ciliary arrays that beat in reverse, generating a specific flow field that facilitates the transport of particles and nutrients to the larva’s surface for subsequent capture. Correspondingly, we design an analogous structure that incorporates a combinatorial arrangement of + and – ciliary bands, as shown in Fig. 5a.

When a synthetic band with this arrangement is exposed to ultrasound, microparticles in close vicinity of the + ciliary band are initially attracted to and traveled along the left ciliary array, then at the center of the + ciliary band are pushed away at velocities of up to 2 mm/s (Supplementary Movie 8). Were this a stand-alone + ciliary band, the trajectories of these particles would have followed the symmetrical vortices, as in Fig. 3a. However, the presence of an adjacent – ciliary band breaks the symmetry of a stand-alone + ciliary band and instead pulls these particles towards the right half of the + ciliary band. Subsequently, in accordance with the source-like flow profile generated by a + ciliary band, these particles move away from the + ciliary band. Given that the strength of the source diminishes with distance from the + ciliary band, the effect of the adjacent sink becomes more prominent as the microparticles progress, causing them to be transported towards the – ciliary band, as shown in Fig. 5b. Figure 5c, d demonstrate the trajectory and velocity behavior of microparticles exposed to the trapping flow field provoked by a trapping ciliary band configuration. As expected, the particle decelerates as it moves away from the + band, then accelerates upon approach towards the – band.

**Fig. 6:**
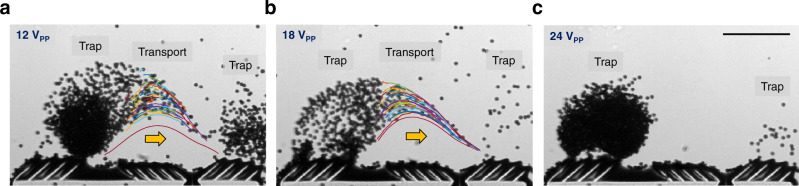
Acoustic power-dependent transport and trapping. Transport and trapping of 10 µm particles (colored trajectories) with maximum transport efficiencies of microparticles traveling from + to – ciliary band were achieved at **a** 12 and **b** 18 volts peak-to-peak (V_PP_). **c** The trapping mode became dominant as excitation (Supplementary Software 4). Scale bar, 200 μm.

We further characterized the trapping and transport mode of the acoustic ciliary bands. A trapping microarchitecture in a bead-solution mixture was subjected to a range of driving voltages. Transport efficacy of microparticles traveling from + to – ciliary band can be regulated by tuning the intensity of the acoustic field (Supplementary Movie 9). Specifically, optimum transport efficiencies along the surface of – ciliary bands were achieved at 12 and 18 V_PP_ (Fig. 6a, b), whereas the trapping mode became dominant as excitation voltage increased to 24 V_PP_, i.e., no particle was transported to the – ciliary band (Fig. 6c). This indicates that the transport efficacy can be tuned by varying the amplitude of the external applied acoustic field (see also Supplementary Fig. 10). Taken together, these results suggest that this biomimetic strategy for the controlled migration of microparticles can be adopted for the development of multifunctional microrobotic systems that can attract, capture, and transport microparticles. Overall, this arrangement of adjacent + and – ciliary bands allow the migration of microparticles from one ciliary band to another. Combined with an efficient capture strategy that further siphons these particles into the body, this transport mechanism can be used to design efficient microrobotic systems that can attract and capture particles of interest from the surrounding flow field.

## Discussion

We developed ultrasound-activated synthetic ciliary bands inspired by the natural ciliary arrangements on the surface of starfish larva. When our planar ciliary bands are angled toward each other (+), fluid is pushed away from the surface on which the cilia are arranged. In contrast, when the cilia are angled away from each other (−), the liquid is forced in toward the bands. We further incorporated these aspects to develop a physical and design principle for acoustic-based microrobots. Although our microrobot’s design enables one-directional motion, we look forward to incorporating superparamagnetic particles such that a combination of ultrasound and magnetic field can be used for propulsion and navigation, respectively^55^. We will focus on the automation and the control part of the microrobotic systems. Alternatively, we plan on developing a hybrid ciliary band in which gas-filled microbubbles are incorporated between individual cilia. Microbubbles of different sizes have different resonance frequencies^47,56^; thus, differently-sized bubbles can be used to selectively activate ciliary bands. With this mechanism, we can develop a frequency-based control unit that will allow our microrobot to be steered using only ultrasound. Future work will also include a thorough interaction of multiple microrobots in ultrasound (see also Supplementary Fig. 11).

We also demonstrate a microparticle transport mechanism analogous to a starfish larva’s feeding mechanism by placing + and – ciliary bands adjacent to each other. This concept can be utilized in lab-on-a-chip systems to realize label-free trapping, fluid mixing and pumping at low Reynolds fluid low, and separation of particles for microfluidics applications. In this study, the angle of our ciliary bands is fixed, however, we plan that a light-activated liquid crystal polymer can be used to dynamically change the orientations of ciliary bands, i.e., switch from + to – arrangements and vice versa, thereby enabling the development of a robotic system that closely mimics its natural counterparts in propulsion and feeding (trapping) mechanisms. Future work will also investigate the trapping and transport of microparticles during propulsion.

The ultrasound cilia and ciliary bands are unique in comparison to natures’ cilia, which produce nonlinear whip-like motion to overcome the reversibility of low-Re flows. Specifically, unlike natural cilia, ultrasound cilia undergo reciprocal motion to produce a flow. This contradiction with the scallop theorem can be attributed to the oscillation frequency of ultrasound cilia that is at least three orders of magnitude higher than its natural counterpart. As revealed by our theoretical analysis, it is this separation of time scales between the (second-order) fluid response and the (firs-order) acoustic actuation that is responsible for the bulk fluid motion, despite the reciprocal motion of cilia. Interestingly, when synthetic cilia were exposed to low-frequency ultrasound between 1 to 100 Hz (see also Supplementary Fig. 4), individual particles demonstrate to and fro motion without any net displacement, further confirming the crucial role of the excitation frequency.

We believe the present work introduces a design space for externally-actuated field-driven microrobots and the engineering of cilia and ciliary bands that are not exclusive to ultrasound-based systems. Engineering cilia that could produce reciprocal motion in order to introduce inertia are much simpler in terms of their fabrication and operation. The concept can be transferred to non-ultrasound methods, for example in magnetic field-driven systems. Recent studies on magnetism-based synthetic cilia mimic the nonreciprocal whip-like beating pattern of biological cilia; however, engineering these devices requires multistep fabrication, and they are challenging to scale down to the microscale. Achieving reciprocal motion in the kHz domain with magnetically-doped engineered cilia may introduce inertia at microscales but may be challenging due to (i) the need to rapidly switch the magnetic field and (ii) magnetic hysteresis, the retention of magnetic alignment after a field is removed. In addition to magnetic-field-driven systems, light-driven liquid crystal-doped polymeric structures have been shown to generate fast movement and to enable rapid switching in the kHz domain, and thus could become a potential candidate mechanism for propulsion via reciprocal motion.

Future work will aim to investigate the oscillation patterns and corresponding flow profiles for cilia with differing geometries, varying the length, width, pitch, angular orientation, and material stiffness. When the microarchitecture is exceptionally soft, the ciliary bands do not retain their shape, i.e., each cilium deforms due to its weight, which resulted in a poor tangential flow along the ciliary array (see also Supplementary Fig. 12 and Supplementary Movie 10). We further investigated the flow profile that results from ciliary array arranged on curved surfaces. Our initial results are consistent with the ciliary array created on a flat surface; hence, the developed ciliary array and bands should operate on curved surfaces as well (see also Supplementary Fig. 13 and Supplementary Movie 11). We will study the performance of our ultrasound-based ciliary bands in terms of fluid flow, propulsion, and particle transport in biologically relevant fluid mediums. Since the attenuation of ultrasound is small in the biological medium, we envision controlling our microrobots in animal models. These soft microarchitectures could become suitable for locally manipulating and administering drugs in the stomach. We plan to track the microrobots in the stomach by incorporating ultrasonic imaging agents in the bulk body and monitoring microrobot behavior using an ultrasound imaging system. The next step also involves investigating their performance in non-Newtonian liquids such as blood, viscoelastic mediums, and shear-thinning gels.

## Methods

### Fabrication method

Ciliary bands were fabricated using a custom-built projection UV photolithography method developed on an inverted microscope (Supplementary Fig. 1). A UV lamp (NIKON, Intensilight C-HGFI) was mounted onto an inverted epi-fluorescence microscope (NIKON, Eclipse Ti) and used to irradiate a high-resolution photomask (CAD/Art Services, Inc.) inserted into the field stop of the microscope. UV light passed through the mask, became focused through a 20x objective, and polymerized the photosensitive mixture (see Materials) downsized by a factor of ~16.3. In exposed regions, the negative photoresist polymerized according to the photomask pattern. The UV exposure time was controlled by an electric shutter system (Vincent Associates, VCM-D1) to fully polymerize the ciliary band and varied between 500–3000 ms at different UV-intensity levels (12.5–100.0%) selected on the UV lamp.

### Materials

The photosensitive polymer mixture used in this project consists of photo-cross-linkable polyethylene glycols (PEGs) and a photo-initiator. The ratios of each component in the mixture were: 50% (v/v) PEG diacrylate with a molecular weight of 700 (PEG 700, Sigma-Aldrich), 30% (v/v) PEG diacrylate with a molecular weight of 258 (PEG 258, Sigma-Aldrich), 15% (v/v) TE buffer (100 TE, Sigma-Aldrich), and 5% (v/v) photo-initiator 2-hydroxy-2-methyl-1-phenyl-propan-1-one (Darocur 1173, Sigma-Aldrich) as photo-initiator. In addition, a droplet (~50 μl) each of fluorescent Rhodamine B (Sigma-Aldrich) and food dye were added to facilitate the focusing of the photomask and to achieve better contrast for the microrobots suspended in DI water.

### Acoustic setup

The acoustic system (Supplementary Fig. 3) was built on a 25 mm × 75 mm ×1 mm glass slide (Menzel) whereon a transducer disc (Murata, 7BB-27-4L0) was attached with epoxy resin (2-K-Epoxidkleber, UHU Schnellfest). Once the ciliary band and other microarchitectures were fabricated and cleaned (IPA, Sigma-Aldrich) on the acoustic device, they were transferred onto the microscope stage. A solution consisting of 10:1 by volume DI water and tracer 5.7 μm particles (Polysciences) was placed on top of the microstructures. A confined “liquid manipulation chamber” was developed by applying a coverslip (22 mm × 22 mm) to the droplet (~150 μl) containing microstructures and tracer particles. The piezo transducer was then connected to the function generator (AFG 3011 C, Tektronix) via an amplifier (Thurlby Thandar Instruments, WA301) to generate acoustic fields with adjustable frequencies and voltages within the liquid. The whole setup was mounted on an inverted microscope.

### Imaging and data analysis

Experiments were performed on a Zeiss Axiovert 200 M inverted microscope equipped with a fluorescent camera (Coolsnap EZ, Photometrics) on its side port and a high-speed camera (CHRONOS 1.4, Kron Technologies) on its top port. Recorded videos were analysed using ImageJ and MATLAB (PIVlab) software.

### Numerical simulation

The numerical simulations reported in this work were conducted using the commercial COMSOL Multiphysics finite element software. For the first-order system of equations, which is indicative of the acoustic response of the system, we sought time-harmonic solutions with a frequency equal to the actuation frequency. In contrast, we sought steady solutions for the second-order components, which represent the time-averaged response of the system. These systems of equations were solved in a sequential manner wherein the first-order solution was used to calculate the forcing terms in the second-order system of equations. We performed 2D simulations on a rectangular domain that was chosen to be sufficiently large to model quiescent fluid around the microswimmer. The oscillating cilia were assigned a nonzero Dirichlet boundary condition for the first-order velocity and a homogeneous Dirichlet boundary condition for the second-order Lagrangian velocity. We employed a triangular mesh with P1-P2 composite elements for the pressure and velocity, where P1 and P2 denote triangular elements with Lagrange polynomials of order 1 and 2, respectively. For both the first- and second-order system of equations, a direct solver was utilized to obtain the solution.

## Supplementary information

Supplementary Information

Description of Additional Supplementary Files

Supplementary Software 1

Supplementary Software 2

Supplementary Software 3

Supplementary Software 4

Supplementary Movie 1

Supplementary Movie 2

Supplementary Movie 3

Supplementary Movie 4

Supplementary Movie 5

Supplementary Movie 6

Supplementary Movie 7

Supplementary Movie 8

Supplementary Movie 9

Supplementary Movie 10

Supplementary Movie 11

## Data Availability

The authors declare that data supporting the findings of this study are available within the paper and its Supplementary Information. Source data are provided with this paper.

## Source data

Source Data
