# Supplementary material for: Ultrasound-activated ciliary bands for microrobotic systems inspired by starfish
Source: Nat Commun. 2021 Nov 9;12:6455. doi: 10.1038/s41467-021-26607-y (PMC8578555; doi:10.1038/s41467-021-26607-y)
Supplement: Supplementary file 1 — Supplementary Information [file 41467_2021_26607_MOESM1_ESM.pdf]

Supplementary Information for

Ultrasound-activated Ciliary Bands for Microrobotic Systems Inspired by Starfish

Cornel Dillinger, Nitesh Nama, and Daniel Ahmed*

Prof. Dr. Daniel Ahmed
Email: dahmed@ethz.ch

Supplementary Figures

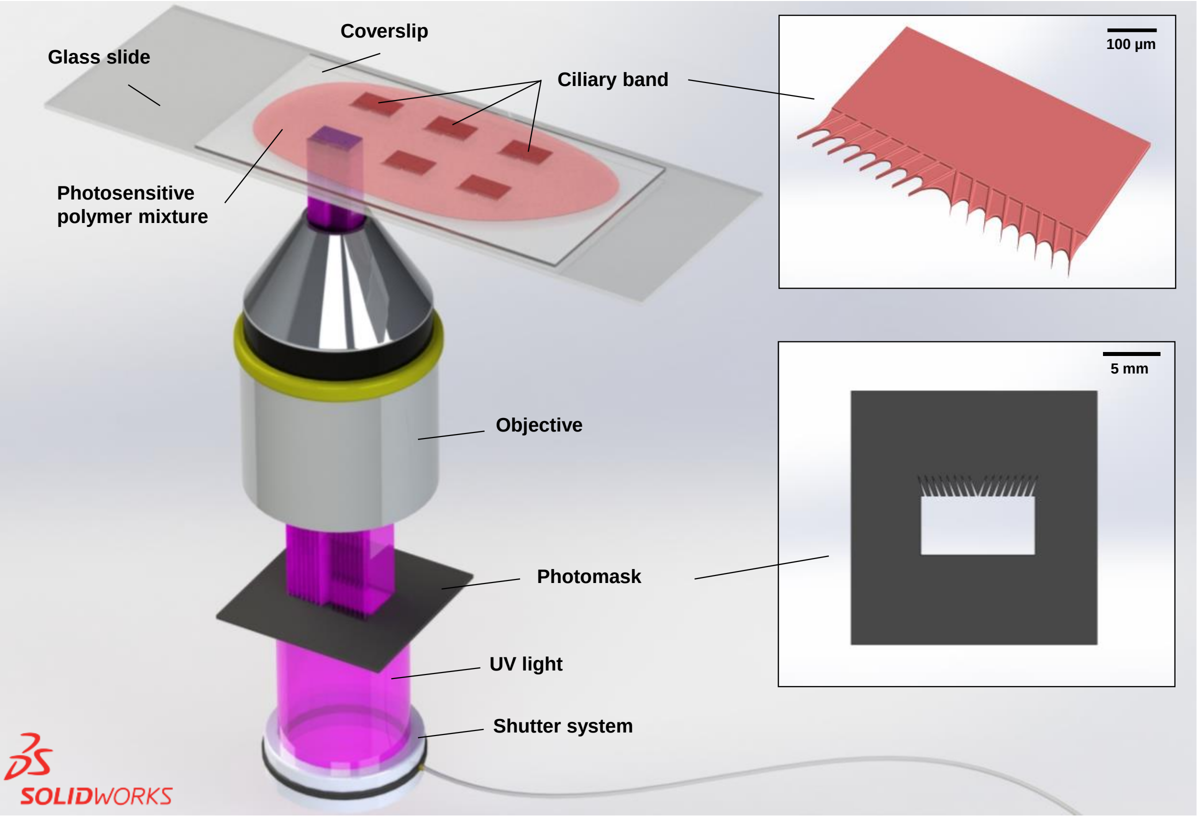

Supplementary Fig. 1 | UV photopolymerization setup. Ciliary bands were fabricated using a custom-built microscope projection UV photolithography process. A UV lamp (NIKON, Intensilight C-HGFI) was mounted onto an inverted epi-fluorescence microscope (NIKON, Eclipse Ti) and used to irradiate a high-resolution photomask (CAD/Art Services, Inc.) inserted into the field stop of the microscope. UV light passed through the mask, became focused through a 20x objective, and polymerized the photosensitive polymer mixture (see Materials) on a glass slide downsized by a factor of ~ 16.3. In exposed regions, the negative photoresistor polymerized according to the photomask pattern. The UV exposure time was controlled by an electric shutter system (Vincent Associates, VCM-D1) to fully polymerize the ciliary band and varied between $500 - 3000$ ms at different UV-intensity levels $(12.5 - 100.0\%)$ selected on the UV lamp.

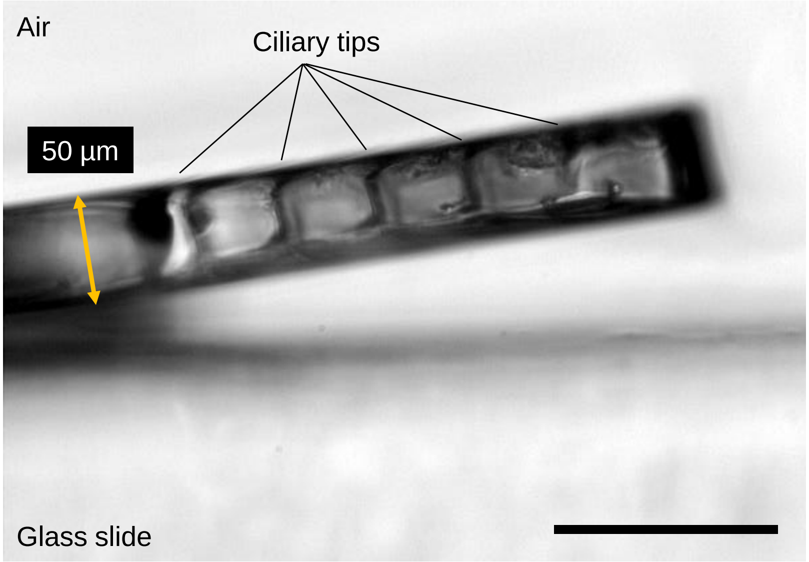

Supplementary Fig. 2 | Side view of a ciliary band. The micrograph shows the cross-sectional view of the ciliary band of height $\sim 50\mu m$. Scale bar, $100\ \mu m$.

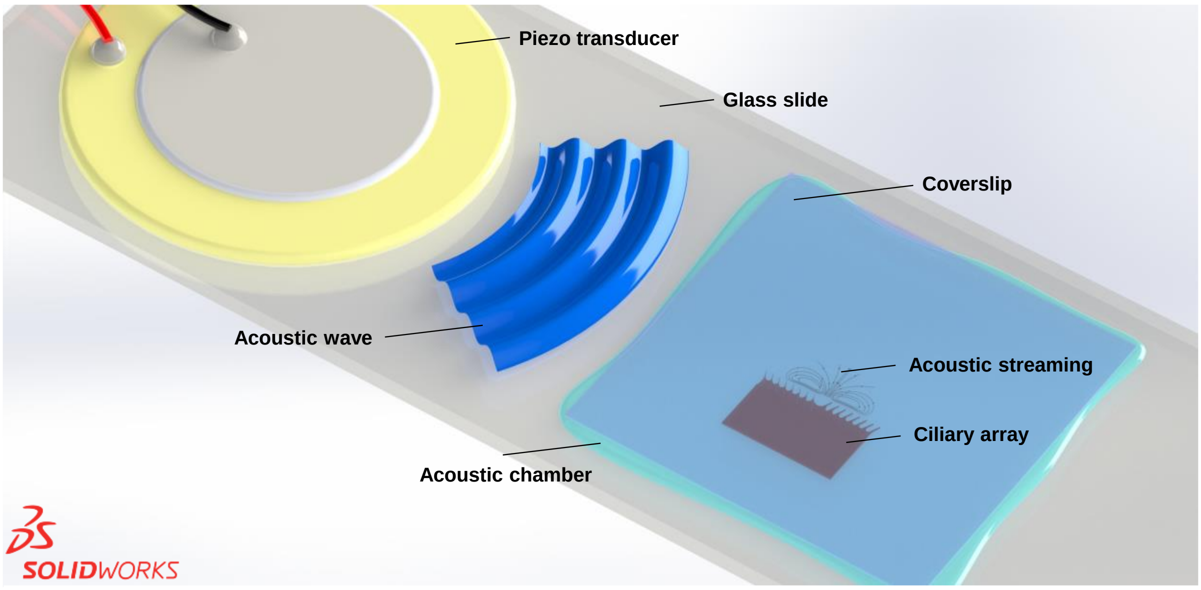

Supplementary Fig. 3 | Acoustic setup. The acoustic system was built on a $25\ \mathrm{mm}\ \mathrm{x}\ 75\ \mathrm{mm}\ \mathrm{x}\ 1\ \mathrm{mm}$ glass slide (Menzel) whereon a transducer disc (Murata, 7BB-27-4L0) was attached with epoxy resin (2-K-Epoxidkleber, UHU Schnellfest). Once the ciliary band and other microarchitectures were fabricated and cleaned (IPA, Sigma-Aldrich) on the acoustic device, they were transferred onto the microscope stage. A solution consisting of $10:1$ by volume DI water and tracer $5.7\ \mu\mathrm{m}$ particles (Polysciences) was placed on top of the microstructures. A confined “liquid manipulation chamber” was developed by applying a coverslip $(22\ \mathrm{mm}\ \mathrm{x}\ 22\ \mathrm{mm})$ to the droplet $(\sim 150\ \mu\mathrm{l})$ containing microstructures and tracer particles. The piezo transducer was then connected to the function generator (AFG 3011C, Tektronix) via an amplifier (Thurlby Thandar Instruments, WA301) to generate acoustic fields with adjustable frequencies and voltages within the liquid. The whole setup was mounted on an inverted microscope.

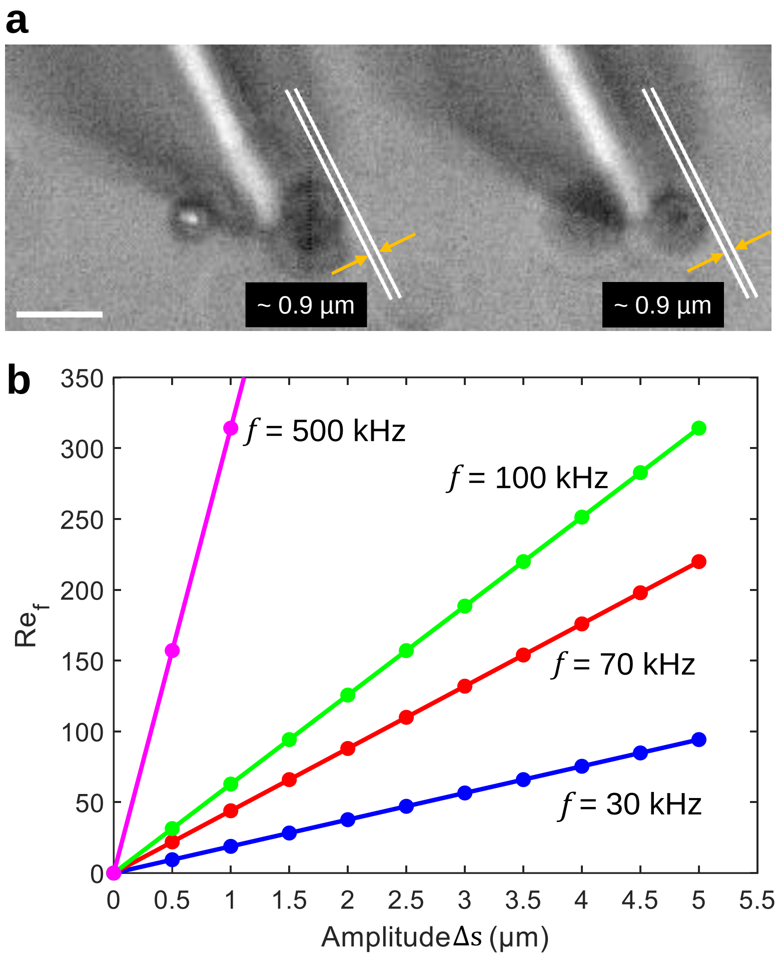

Supplementary Fig. 4 | Oscillation characteristics of the ciliary bands. a. Optical image demonstrates the small-amplitude oscillation of the ciliary bands in ultrasound. The image shows the oscillation amplitude, $\Delta s \approx 0.9\,\mu m$, of the ciliary band at $68.5\,kHz$ and $20\ V_{PP}$ (see also Movie S1). Scale bar, $10\ \mu m$. b. Measured Reynolds number ($Re_f = 2\pi f \Delta s L/\nu$) versus oscillation amplitude Δs of the cilia tip at 30 (blue), 70 (red), 100 (green), and $500\ kHz$ (magenta).

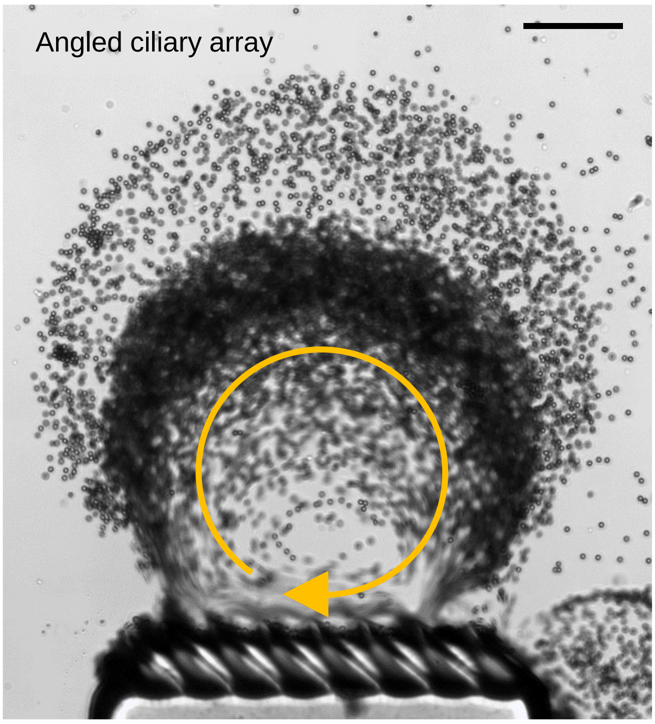

Supplementary Fig. 5 | Vortex generation in an angled ciliary array. A single ciliary array produces a clockwise motion at $68.5\ \mathrm{kHz}$ and $20.0\ \mathrm{V_{PP}}$ (see also Movie S3). Scale bar, $100\ \mu\mathrm{m}$

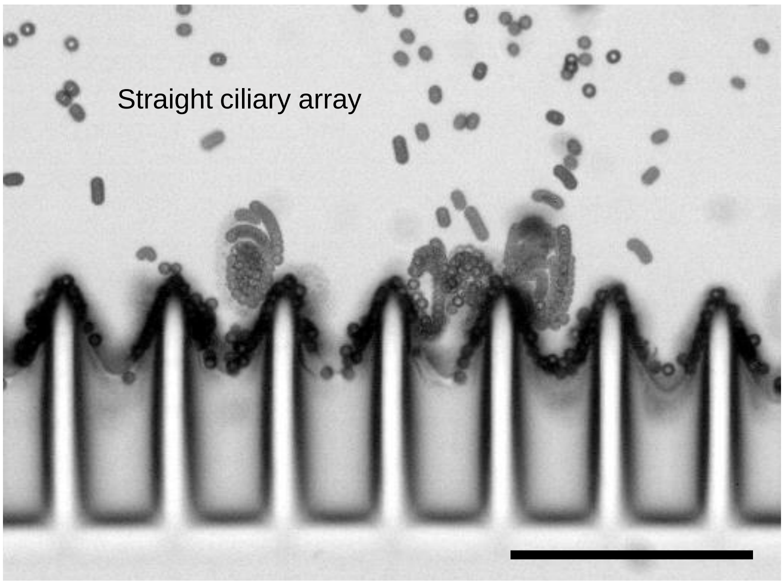

Supplementary Fig. 6 | Localized vortices developed between straight ciliary array. The control experiment demonstrates counter-rotating vortices developed at tips of the straight ciliary array at excitation frequency and amplitude of $68.5\ \mathrm{kHz}$ and $20\ \mathrm{V_{PP}}$, respectively. The stacked images captured at 2871 fps (frames per second) show no tangential movement of the tracer particles (see also Movie S5). Scale bar, $100\ \mu\mathrm{m}$.

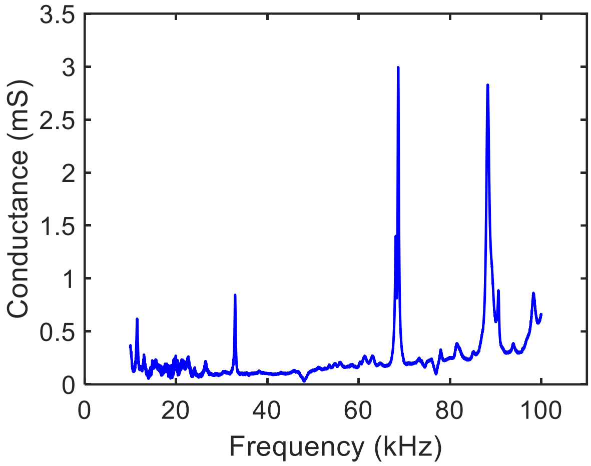

Supplementary Fig. 7 | Resonance characteristics of the piezoelectric transducer. Admittance measurement (Real part of admittance = conductance) of the piezo transducer coupled with a glass slide using an impedance analyzer (SinePhase, Impedance Analyzer 16777k). The experimental setup showed resonances at ~ 32.9, ~ 68.7, and ~ 88.3 kHz.

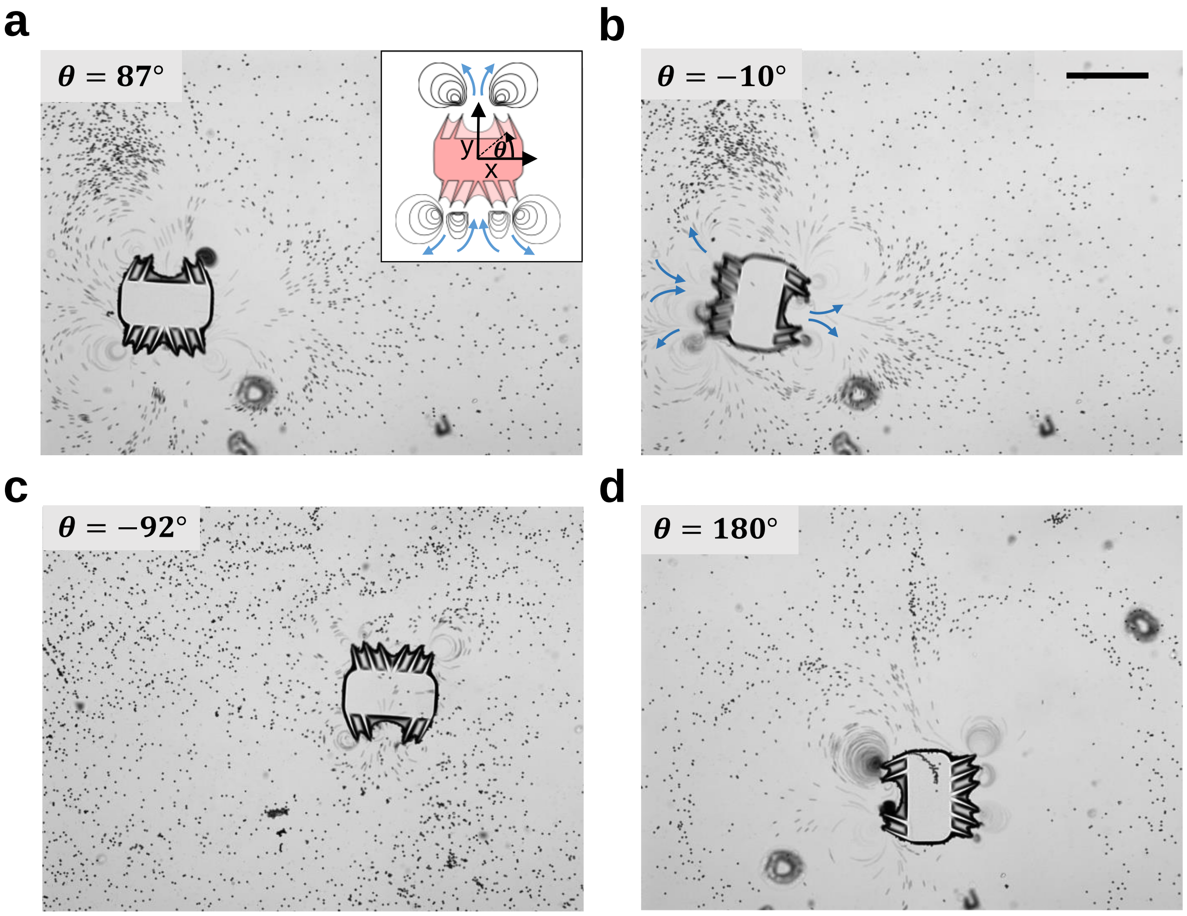

Supplementary Fig. 8 | Orientation independent flow field of the microswimmer with respect to the direction of the background acoustic field. The acoustic streaming profiles effected by the + and – ciliary bands were conserved across different microrobot orientations as demonstrated in a., b., c, and d. Scale bar, 250 μm.

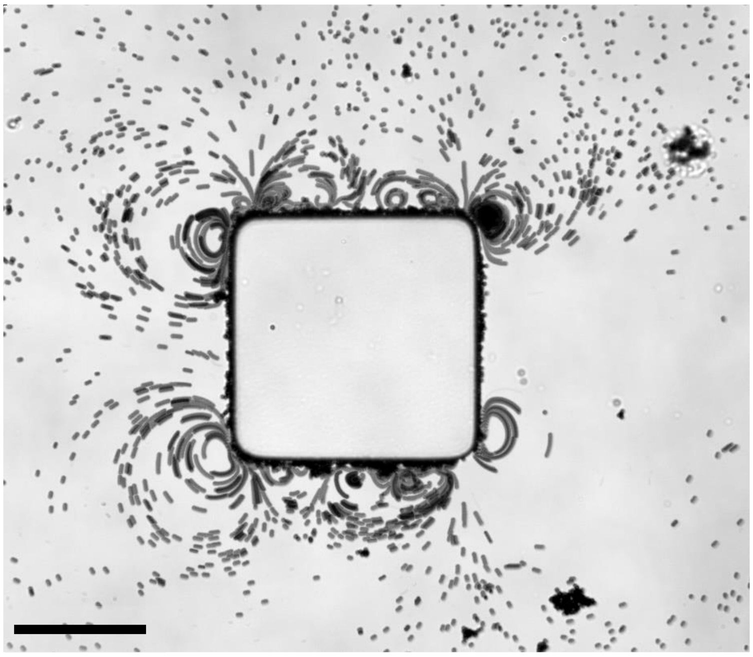

Supplementary Fig. 9 | Acoustic response of a non-ciliated microstructure. Non-ciliated microstructure induces weak streaming when exposed to ultrasound with frequency and amplitude of $68.5\ \mathrm{kHz}$ and $20\ \mathrm{V_{PP}}$, respectively. No net displacement of the microstructure with similar dimensions as the ciliated microrobot was observed. Scale bar, $200\ \mu\mathrm{m}$.

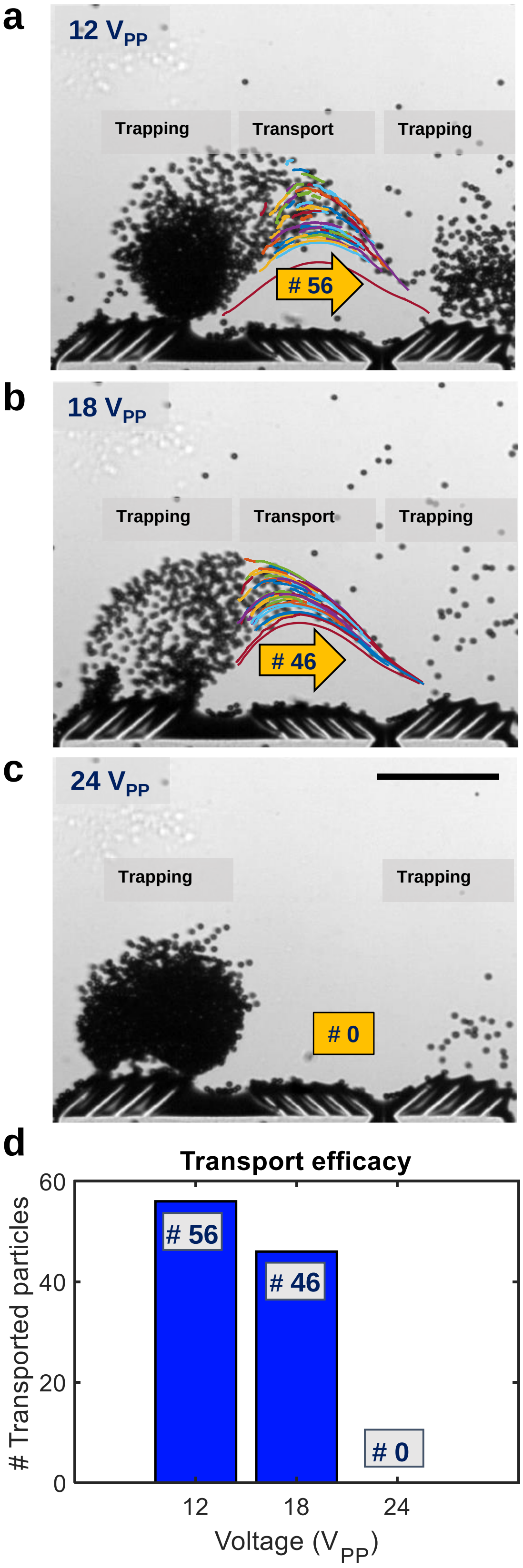

Supplementary Fig. 10 | Acoustic power-dependent transport and trapping mode. Transport of 10 µm microparticles travelling from + to – ciliary band were achieved at a. 12 and b. 18 V_{PP}. c. The trapping mode became dominant as excitation voltage increased to 24 V_{PP}, i.e., no particle was transported to the – ciliary band over 3000 frames at 2871 fps (see also Movie S9). d. Maximal transport efficiencies were achieved at 12 and 18 V_{PP}, with 56 and 46 particles respectively tracked over 3000 frames at 2871 fps. Scale bar, 200 µm.

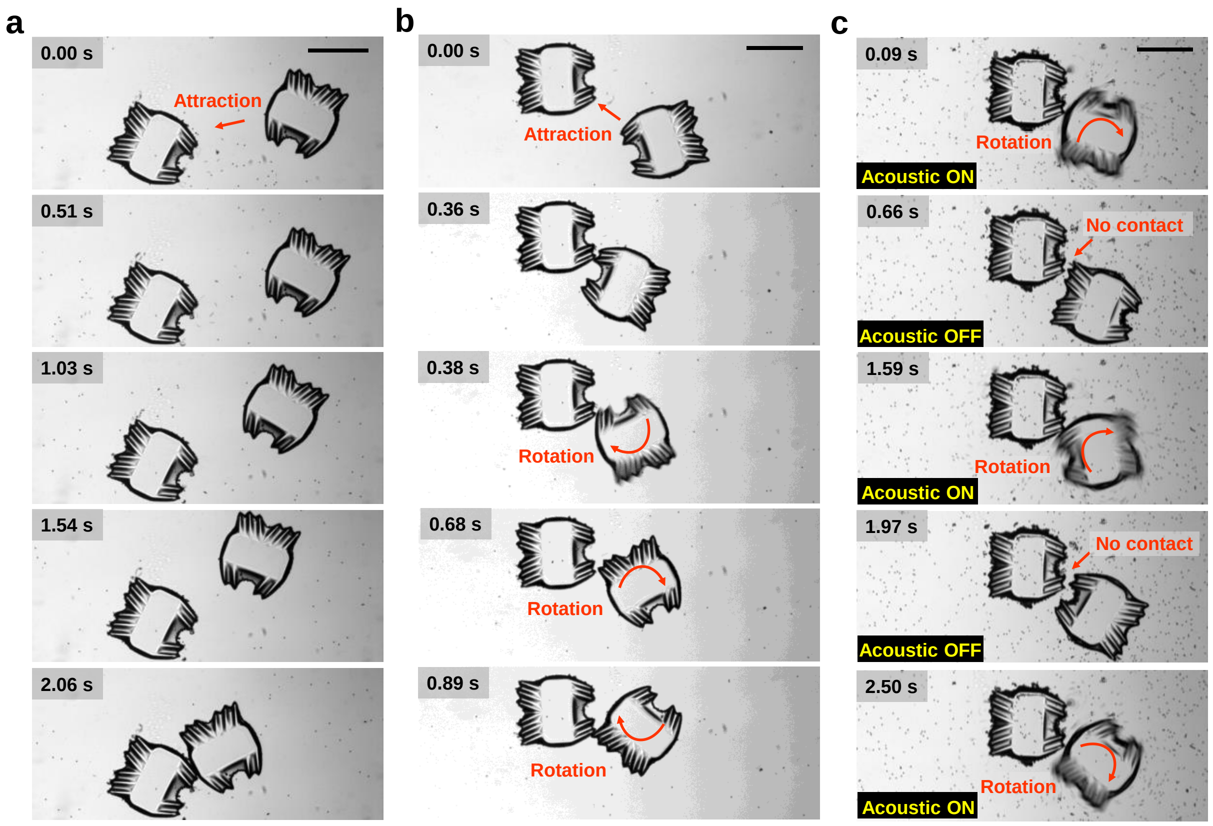

Supplementary Fig. 11 | Interaction of two microrobots in ultrasound. a. A fixed microrobot (left) attracts a second approaching microrobot ($f = 68.1$ kHz and 27.0 V_{PP}). b. A fixed microrobot attracts a second approaching microrobot and rotates it in its flow field ($f = 68.1$ kHz and 22.5 V_{PP}). c. A fixed microrobot under acoustic stimulation rotates a second microrobot in its vortex, losing contact when the stimulation is turned off. Scale bars, 250 μm.

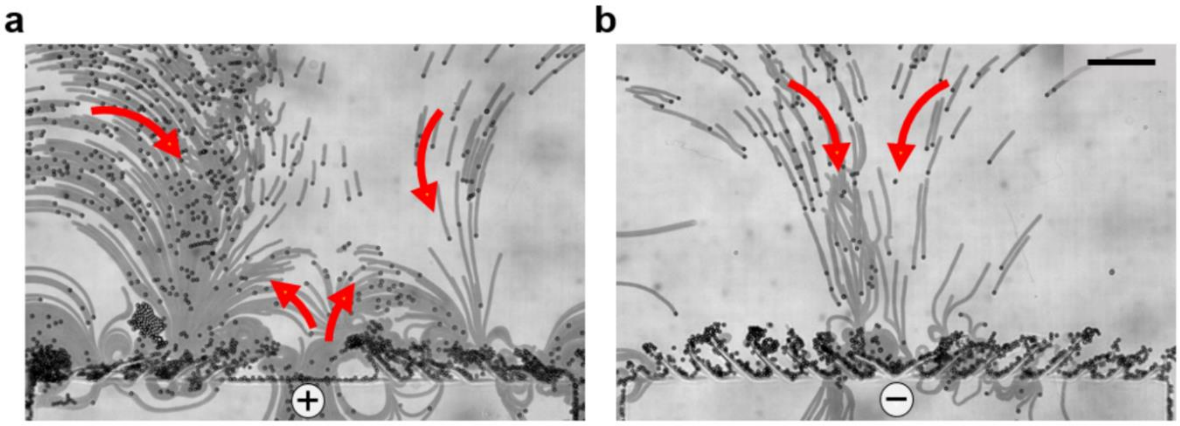

Supplementary Fig. 12 | Acoustic streaming in soft ciliary bands. Image sequences demonstrating the weak acoustic streaming characteristics on a. + and b. – ciliary bands excited at $68.5\ \mathrm{kHz}$ and $20.0\ V_{PP}$ respectively, (see also Movie S10). We used a higher numerical aperture objective (Nikon 20x, CFI Plan APO VC, N.A.= 0.75) to fabricate high-resolution ciliary bands without web-like structures. The cilia tend to collapse under their own weight and cannot be activated to oscillate. Scale bar, $100\ \mu\mathrm{m}$.

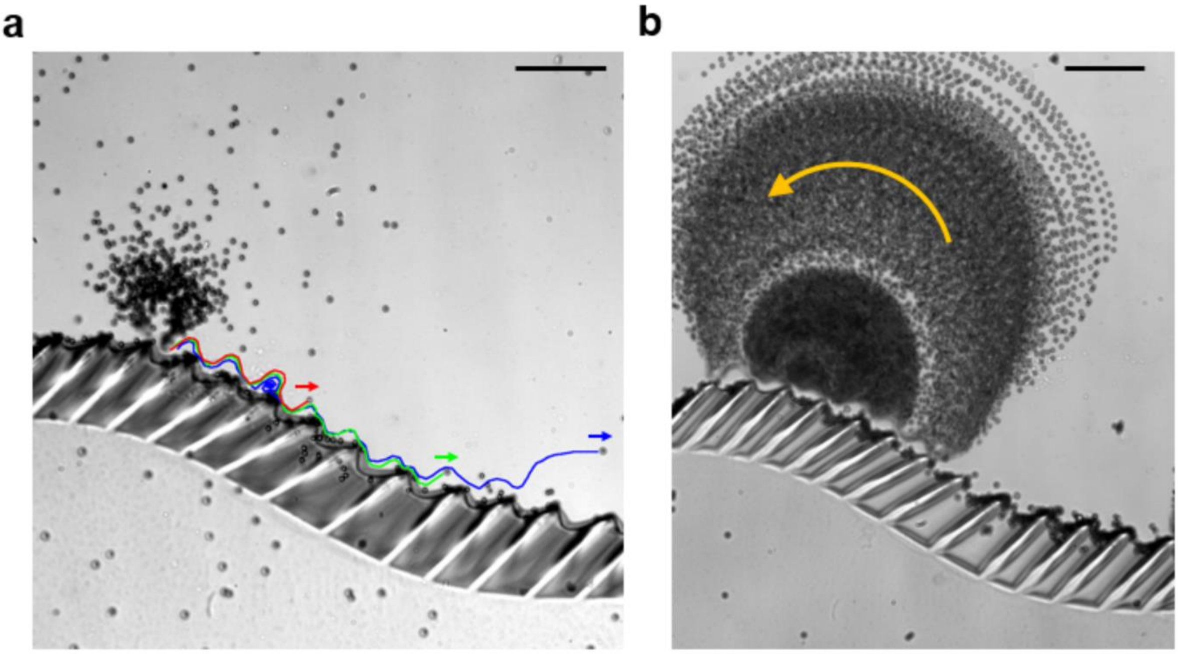

Supplementary Fig. 13 | Particle transport along curved ciliary array. a. Individual tracer particles hop from one ciliary tip to the next on a curved ciliary array, i.e., tangential flow along the capillary was observed, as indicated by red, green, and blue trajectories, travelling left-to-right. The tracers follow the ciliary array continuously as long as the inter-tip spacing remains approximately uniform. The ultrasound excitation frequency was $68.5\ \mathrm{kHz}$ and the amplitude $22.5\ \mathrm{V_{PP}}$ (see also Movie S11). b. A counter-clockwise (CCW) vortex was also produced along the curved ciliary array under similar experimental conditions as described in a. Scale bars, $100\ \mu\mathrm{m}$.

Supplementary Notes

Theory

Here, we describe the theoretical formulation employed in the current work to investigate the flow patterns around microswimmer. The fluid response is governed by the standard Navier-Stokes equations for a linear, viscous compressible fluid:

$$\frac{\partial \rho}{\partial t} + \nabla \cdot (\rho \mathbf{v}) = 0 \ , \tag{S1}$$

$$\rho \frac{\partial \mathbf{v}}{\partial t} + \rho(\mathbf{v} \cdot \nabla)\mathbf{v} = -\nabla p + \mu \nabla^2 \mathbf{v} + (\mu_b + \frac{1}{3}\mu)\nabla(\nabla \cdot \mathbf{v}), \tag{S2}$$

where ρ is the mass density of the fluid, p is the fluid pressure, and μ and μ_b are the shear and the bulk dynamic viscosities, respectively. Eq. (S1) and (S2) need to be supplemented with a constitutive relation linking the pressure to the fluid density. Following our prior work [1,2], we assume a linear relation between pressure and density as:

$$p = c_0^2 \rho,$$

where c_0 is the speed of sound in the fluid under quiescent conditions. Direct simulations of this system of equations (with appropriate boundary conditions) poses significant numerical challenges due to the widely separated time scales of the acoustic actuation and the consequent acoustic streaming. Therefore, we follow a perturbation approach to split the flow variables into their first- and second-order components as:

$$\mathbf{v} = \mathbf{v_0} + \varepsilon \mathbf{v_1} + \varepsilon^2 \mathbf{v_2} + O(\varepsilon^3) + \cdots ,$$

$$p = p_0 + \varepsilon p_1 + \varepsilon^2 p_2 + O(\varepsilon^3) + \cdots ,$$

$$\rho = \rho_0 + \varepsilon \rho_1 + \varepsilon^2 \rho_2 + O(\varepsilon^3) + \cdots , \tag{S3}$$

where ε is a non-dimensional smallness parameter. Substitution of (S3) in (S1) and (S2), and segregation of first-order terms yields a first-order system of equations:

$$\frac{\partial \rho_1}{\partial t} + \rho_0(\nabla \cdot \mathbf{v_1}) = 0, \tag{S4}$$

$$\rho_0 \frac{\partial \mathbf{v}_1}{\partial t} = -\nabla p_1 + \mu \nabla^2 \mathbf{v_1} + (\mu_b + \tfrac{1}{3}\mu)\nabla(\nabla \cdot \mathbf{v_1}). \qquad \text{(S5)}$$

Following the same procedure for the second-order terms, and a subsequent time-averaging over a period of oscillation, yields the second-order system of equations:

$$\langle \frac{\partial \rho_2}{\partial t} \rangle + \rho_0 \nabla \cdot \langle \mathbf{v}_2 \rangle = -\nabla \cdot \langle \rho_1 \mathbf{v}_1 \rangle, \qquad \text{(S6)}$$

$$\rho_0 \langle \frac{\partial \mathbf{v_2}}{\partial t} \rangle + \langle \rho_1 \frac{\partial \mathbf{v}_1}{\partial t} \rangle + \rho_0 \langle (\mathbf{v}_1 \cdot \nabla) \mathbf{v}_1 \rangle = -\nabla p_2 + \mu \nabla^2 \mathbf{v}_2 + (\mu_b + \tfrac{1}{3}\mu)\nabla(\nabla \cdot \mathbf{v}_2). \quad \text{(S7)}$$

We have retained the terms associated with the bulk viscosity in our analysis to fully account for viscous attenuation both within and outside the boundary layer. The above system of equations, with appropriate boundary conditions, yields the Eulerian descriptions of fluid velocity and pressure at the first- and second-order. However, for a direct correspondence with the particle velocity observed in the experiments, these descriptions need to be subsequently augmented with the definition of Stokes drift to obtain the Lagrangian fluid velocity. Alternatively, as discussed in [2], a similar system of equation as above can be derived directly in terms of the Lagrangian fluid velocity to avoid the need to employ the notion of Stokes drag. We refer the reader to our prior work [3] for further discussion of the governing equations and boundary conditions.

Scaling with frequency and fluid viscosity:

To better understand the effect of actuation frequency and fluid viscosity on the flow profiles observed in our system, we follow the dimensional arguments similar to those reported by Ovchinnikov et al [4]. We note that, from a mathematical perspective, the scenario where the ciliary bands oscillate in a stationary fluid is the same as the one where the ciliary bands are fixed while the fluid oscillates, i.e., the two scenarios represent a simple change of reference frame. Therefore, for this analysis, the fluid is assumed to be oscillating with a velocity amplitude v_∞, while the ciliary bands are kept fixed. Specifically, for an individual (straight) cilia, the first-order perturbations in the velocity field can be expressed as[4]

$$\mathbf{v_1}\,(r) \;=\; v_\infty \left(\frac{\delta}{a}\right)^{n-1} \Phi_\alpha\left(\frac{r}{\delta}\right), \qquad \text{(S8)}$$

where Φ_α is a unique (dimesionless) function that depends only on the cilia tip angle (α), a is the linear dimension of the body, and $n = \frac{\pi}{(2\pi-\alpha)}$ is an exponent depending on cilia tip angle (Fig. S14). Similarly, the scaling of the streaming velocity is obtained as

$$\mathbf{v_0}\,(r) = \frac{v_\infty^2}{\nu} \frac{\delta^{2n-1}}{a^{2n-2}} \mathrm{H}_\alpha\left(\frac{r}{\delta}\right), \qquad \text{(S9)}$$

where H_α is another dimensionless function. Here, the frequency scaling of the acoustic streaming around a cilia can be obtained by substituting the expression of viscous boundary layer thickness $\delta = \sqrt{\nu/(\pi f)}$ in the above expression.

We remark that while our system represents a combination of the multiple (tilted) cilia, the overall flow profile can be viewed as a combination of flow profiles generated by several individual cilia. Therefore, these expressions are instructive of the frequency and viscosity scaling governing our system.

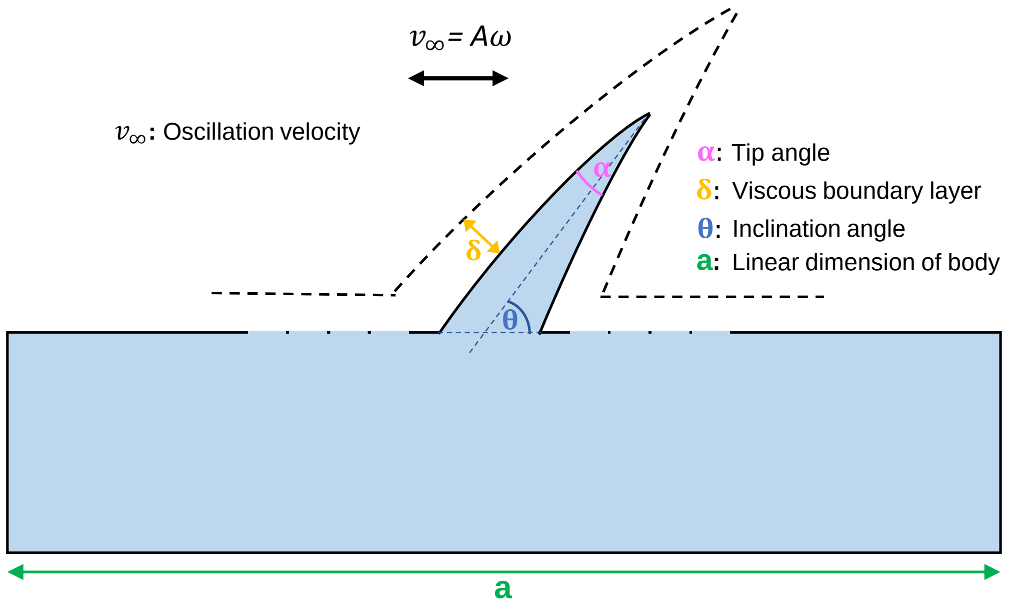

Schematic illustrating the various geometrical parameters and length scales for a single cilia.

Supplementary References

1. Nama, N., Huang, P. H., Huang, T. J. & Costanzo, F. Investigation of acoustic streaming patterns around oscillating sharp edges. *Lab Chip* (2014) doi:10.1039/c4lc00191e.

2. Nama, N., Huang, T. J. & Costanzo, F. Acoustic streaming: an arbitrary Lagrangian--Eulerian perspective. *J. Fluid Mech.* 825, 600–630 (2017).

3. Nama, N., Huang, P.-H., Huang, T. J. & Costanzo, F. Investigation of micromixing by acoustically oscillated sharp-edges. *Biomicrofluidics* 10, 24124 (2016).

4. Ovchinnikov, M., Zhou, J. and Yalamanchili, S.. Acoustic streaming of a sharp edge. The Journal of the Acoustical Society of America (2014), 136(1), pp.22-29
