# Supplementary material for: Ultrasound-activated ciliary bands for microrobotic systems inspired by starfish
Source: Nat Commun. 2021 Nov 9;12:6455. doi: 10.1038/s41467-021-26607-y (PMC8578555; doi:10.1038/s41467-021-26607-y)
Supplement: Supplementary file 2 — Description of Additional Supplementary Files [file 41467_2021_26607_MOESM2_ESM.pdf]

## Description of Additional Supplementary Files

File Name: Supplementary Movie 1
Description: The oscillation amplitude of the ciliary array is visualized when acoustic was switched on and off at excitation frequency and amplitude of $\mathbf{68.5\ kHz}$ and $\mathbf{20\ V_{PP}}$, respectively.

File Name: Supplementary Movie 2
Description: Tangential streaming along an angled oscillating ciliary array at excitation frequency and amplitude of $\mathbf{33.7\ kHz}$ and $\mathbf{5\ V_{PP}}$, respectively

File Name: Supplementary Movie 3
Description: Acoustic streaming induced by uniformly arranged cilia. A single ciliary array produces a clockwise motion of microparticles at an acoustic excitation of $\boldsymbol{f = 68.5\ \mathrm{kHz\ and}\ 20.0\ V_{PP}}$.

File Name: Supplementary Movie 4
Description: Acoustic streaming of a + and – ciliary band at excitation frequency and amplitude of $\mathbf{68.5\ kHz}$ and $\mathbf{20\ V_{PP}}$, respectively.

File Name: Supplementary Movie 5
Description: Acoustic steaming of a straight (non-angulated) ciliary band at excitation frequency and amplitude of $\mathbf{68.5\ kHz}$ and $\mathbf{20\ V_{PP}}$, respectively.

File Name: Supplementary Movie 6
Description: A bioinspired microrobot is developed by arranging + and – ciliary bands opposite to each other. The microrobot executing translational motion with a mean velocity of $\mathbf{v \approx 2.6\ mm/s}$ at excitation frequency and amplitude of $\mathbf{68.8\ kHz}$ and $\mathbf{20\ V_{PP}}$, respectively.

File Name: Supplementary Movie 7
Description: Acoustic streaming profile of the bioinspired microrobot at excitation frequency and amplitude of $\mathbf{68.8\ kHz}$ and $\mathbf{20\ V_{PP}}$, respectively.

File Name: Supplementary Movie 8
Description: A bioinspired trapping mechanism is developed based on a combinatorial arrangement of a + and – configuration of ciliary bands at excitation frequency and amplitude of $\mathbf{68.5\ kHz}$ and $\mathbf{20\ V_{PP}}$, respectively. This configuration exhibits the capability to trap and transport neighboring $\mathbf{10\ \mu m}$ microparticles.

File Name: Supplementary Movie 9
Description: Power-dependent particle transport efficacy. The transport efficacy is highest at 12-18 $\mathbf{V_{PP}}$, where 56 and 46 tracked 10-µm particle trajectories indicate transport. At 24 $\mathbf{V_{PP}}$ no particles are transported.

File Name: Supplementary Movie 10
Description: Weak acoustic streaming of high-resolution ciliary bands without web-like structure at excitation frequency and amplitude of $\mathbf{68.5\ kHz}$ and $\mathbf{20\ V_{PP}}$, respectively.

File Name: Supplementary Movie 11
Description: Ciliary array arranged on a curved surface excited at frequency and amplitude of $\mathbf{68.4\ kHz}$ and $\mathbf{22.5\ V_{PP}}$. The 6-µm particles tangentially hop from one ciliary tip to the adjacent one as long as the inter-tip distance remains approximately the same.

File Name: Supplementary Software 1

Description: MATLAB code processing Source Data 1 for the representation of the tangential velocity plot in Fig. 2b.

File Name: Supplementary Software 2

Description: MATLAB codes processing Source Data 2 for the representation of the vertical velocity of a plus and a minus ciliary band in Fig. 3c and 3d.

File Name: Supplementary Software 3

Description: MATLAB code processing Source Data 3 for the representation of the trapping characteristics of a trapping ciliary band in Fig. 5c and 5d.

File Name: Supplementary Software 4

Description: MATLAB code tracking the trapped flow tracers seen in videos of Supplementary Movie 9. Results are demonstrated in Fig. 6.
